# Sterol O-Acyltransferase 1 (*SOAT1*): A Genetic Modifier of Niemann-Pick Disease, Type C1

**DOI:** 10.3390/ijms25084217

**Published:** 2024-04-11

**Authors:** Nicole Y. Farhat, Derek Alexander, Kyli McKee, James Iben, Jorge L. Rodriguez-Gil, Christopher A. Wassif, Niamh X. Cawley, William E. Balch, Forbes D. Porter

**Affiliations:** 1 Division of Translational Medicine, Eunice Kennedy Shriver National Institute of Child Health and Human Development, National Institutes of Health, Bethesda, MD 20892, USA; nicole.farhat@nih.gov (N.Y.F.); derek.alexander@nih.gov (D.A.); kyli.a.mckee@gmail.com (K.M.); christopher.wassif@astrazeneca.com (C.A.W.); cawleyn@mail.nih.gov (N.X.C.); 2 Molecular Genomics Core, Eunice Kennedy Shriver National Institute of Child Health and Human Development, National Institutes of Health, Bethesda, MD 20892, USA; james.iben@nih.gov; 3 Division of Medical Genetics, Division of Neonatal and Developmental Medicine, Department of Pediatrics, Stanford University, Palo Alto, CA 94304, USA; jrgil@stanford.edu; 4 Department of Molecular Medicine, Scripps Research, La Jolla, CA 92037, USA; webalch@scripps.edu

**Keywords:** Niemann-Pick disease, type C1, NPC1, sterol O-acyltransferase 1, SOAT1, ACAT1, genetic modifiers, neurodegeneration

## Abstract

Niemann-Pick disease type C1 (NPC1) is a lysosomal disorder due to impaired intracellular cholesterol transport out of the endolysosomal compartment.. Marked heterogeneity has been observed in individuals with the same *NPC1* genotype, thus suggesting a significant effect of modifier genes. Prior work demonstrated that decreased SOAT1 activity decreased disease severity in an NPC1 mouse model. Thus, we hypothesized that a polymorphism associated with decreased SOAT1 expression might influence the NPC1 phenotype. Phenotyping and genomic sequencing of 117 individuals with NPC1 was performed as part of a Natural History trial. Phenotyping included determination of disease severity and disease burden. Significant clinical heterogeneity is present in individuals homozygous for the *NPC1^I1061T^* variant and in siblings. Analysis of the *SOAT1* polymorphism, rs1044925 (A>C), showed a significant association of the C-allele with earlier age of neurological onset. The C-allele may be associated with a higher Annualized Severity Index Score as well as increased frequency of liver disease and seizures. A polymorphism associated with decreased expression of *SOAT1* appears to be a genetic modifier of the NPC1 phenotype. This finding is consistent with prior data showing decreased phenotypic severity in *Npc1-/-:Soat1-/-* mice and supports efforts to investigate the potential of SOAT1 inhibitors as a potential therapy for NPC1.

## 1. Introduction

The phenotypes associated with genetic diseases often show incomplete penetrance and variable expressivity. For specific disorders, much of this phenotypic heterogeneity may be due to specific pathological variants in the causative gene that result in variable levels of residual protein function. However, phenotypic heterogeneity is also observed in individuals with the same genotype. An example of this would be incomplete penetrance and variable expressivity manifested in sibling pairs. Although environmental differences likely contribute, genetic modifiers also play a role. The concept of genetic modifiers contributing to human disease variability was initially proposed by Haldane in 1941 [1]. Genetic modifiers can either increase or decrease phenotypic severity; thus, their identification could help provide guardians and patients with prognostic information. In addition, identification of genetic modifiers that alleviate disease severity may provide insight into potential therapeutic approaches.

It is well-established that modifiers may significantly impact the phenotype of rare genetic disorders. Genomic databases consisting of healthy individuals have led to the identification of resilient individuals who, based on their genome sequence, would have been expected to manifest a severe childhood disorder [2,3]. Identification of genes that function as phenotypic modifiers for rare diseases has proven to be difficult, likely due to quantitatively defining the phenotype and statistical constraints due to the rarity of these diseases [4]. Underscoring the difficulty of identifying genetic modifiers, a 2020 bibliographic review by Rahit et al. [5] only identified 24 genes that appear to function as genetic modifiers for rare Mendelian disorders. In this paper, we demonstrate the use of a hypothesis-based approach to characterize a potential genetic modifier of Niemann-Pick disease, type C1.

Niemann-Pick disease, type C1 (NPC1, MIM 257220) is an autosomal recessive, inborn error of intracellular cholesterol transport due to impaired function of NPC1. A similar, but much rarer disorder, Niemann-Pick disease, type C2 (NPC2, MIM 607625), is caused by impaired function of NPC2. NPC2 is a small ~25–27 kDa luminal lysosomal protein and NPC1 is a large 142 kDa (calculated) transmembrane protein localized in the endolysosomal membrane. NPC1 and NPC2 are encoded by *NPC1* on chromosome 18q11 and *NPC2* on chromosome14q24. These two proteins, NPC1 and NPC2, act in concert to move cholesterol from the endolysosomal system and make it bioavailable to the cell. Cholesterol esters contained in lipoprotein particles, such as low-density lipoprotein, enter the cell via receptor-mediated endocytosis. The cholesterol esters are then de-esterified via lysosomal acid lipase (LAL). After removal of the fatty acid via LAL, the unesterified cholesterol is transported by NPC2 to the N-terminal domain of NPC1. NPC1 then facilitates the transit of cholesterol from the endolysosomal lumen to the cytoplasmic face of the endolysosomal membrane where it then can be distributed to other cellular membranes and become functionally bioavailable [6]. Thus, in either NPC1 or NPC2 disease, biallelic variants of either *NPC1* or *NPC2* lead to both endolysosomal storage of unesterified cholesterol and a corresponding deficiency of cellular bioavailable cholesterol. Increased expression of SREBP2 regulated genes, which increases both endogenous synthesis and exogenous cholesterol uptake, is evidence of the functional cholesterol deficiency, even though total cellular cholesterol may be increased [7].

NPC1 is an ultrarare disease with an incidence of the classical disease estimated to be in the order of 1/110,000 [8]. Classically, NPC1 was diagnosed via cellular staining with filipin, a fluorescent compound that specifically labels unesterified cholesterol; however, filipin staining of patient fibroblasts has been supplanted by plasma-based tests measuring levels of cholestane-3β,5α,6β-triol, N-(3β,5α,6β-trihydroxy-cholan-24-oyl)glycine or N-palmitoyl-O-phosphocholineserine/lysosphingomyelin-509 [9,10]. NPC1 can also be diagnosed by sequencing *NPC1*. Molecular diagnosis has been facilitated by inclusion of *NPC1* on sign/symptom gene panels as well as exome/genome sequencing. It is hoped that improved diagnostic modalities will decrease the 4–5-year diagnostic delay that has been observed [10].

Infants with liver disease often present with cholestatic jaundice. Although this can be severe and lead to diagnosis of NPC1, it is often transient and resolves with time. Onset of the neurological signs and symptoms typically follows hepatic disease and has an insidious progression. The NPC1 neurological phenotype is characterized by progressive neurological dysfunction which includes supranuclear vertical gaze palsy, cerebellar ataxia and cognitive impairment. However, the NPC1 phenotype is heterogeneous both with respect to age of neurological onset and the specific sign/symptom complex manifested by individuals [8,11]. Clinically it has been recognized that the NPC1 phenotype can vary between siblings and individuals with the same *NPC1* genotype. Case reports have recently been summarized by Las Heras et al. [12]. Clinical data also suggests that ApoE isotype can modify the NPC1 phenotype. Specifically, in a small case series, the ApoE4 isoform is associated with increased neuropathological findings [13] and earlier onset of neurological signs/symptoms [14]. Miglustat has been shown to be clinically effective in slowing neurological disease progression [15,16,17] and extending survival [18]. Although miglustat is approved for the treatment of NPC in most countries, it is only available off-label in the United States. Currently, there are no FDA-approved therapies for NPC1. Thus, identification of genes/metabolic processes that modify the NPC1 neurological phenotype could provide insight into novel therapeutic approaches.

Multiple studies using different experimental approaches with NPC1 models have provided evidence for genetic modifiers of *NPC1*. The Sturley research group, using an NPC1 yeast model, has identified 12 pathways and 13 genes in a genome-wide, conditional synthetic lethality screen [19]. These data, in combination with a high-throughput drug screening by Pipalia et al. [20], contributed to identification of histone deacetylase inhibition as a potential therapy for NPC1. High-throughput drug screens have shown that modulation of NPC1 activity by either protein stabilization or upregulation of *NPC1* expression can decrease unesterified cholesterol storage. These include HDAC-inhibitor-mediated stabilization of variant NPC1 proteins [20] and alexidine-mediated increased NPC1 expression [21]. RNAi [22] and CRISPRi screens [23] identified *TMEM97* and *SNX13*, respectively, as genes that modify unesterified cholesterol storage in cells deficient for NPC1 function. Genetic background in *Npc1* mutant mice can significantly influence the severity of the NPC1 phenotype. Homozygous *Npc1* mutant mice on a C57Bl/6J background have a more severe phenotype compared to the same mutant alleles on a Balb/cJ background [24,25,26]. Rodriguez-Gil et al. [25] performed a QTL analysis for lifespan between these two different strains, and found significant evidence for linkage to markers on mouse chromosomes 1 and 7. Similarly, Zhang and Erickson [27] observed evidence of a phenotypic modifier on mouse chromosome 19. Multiple studies have evaluated potential genetic modifiers in double-mutant mice. Disruption of genes encoding enzymes involved in glycosphingolipid synthesis, GM2/GD2 synthase [28] and GM3 synthase [29], decrease neuropathology in *Npc1* mutant mice. Recognition that decreased glycosphingolipid synthesis was associated with phenotypic improvement contributed to the development of miglustat as a therapy for NPC1. Although neuroinflammation is a predominant neuropathological finding in NPC1, modulation of genes expressed as part of the immune response in general has had relatively limited impact on the survival of *Npc1* mutant mice. Examples include disruption of Il6 [30], C1qa and C3 [31] and Ccl3 [32]. In contrast, a recent work suggests that modulation of either STING or IRF3 may have beneficial effects in NPC1 [33,34]. Decreased expression of tau (*Mapt*) and amyloid precursor protein (*App*) increased phenotypic severity [35,36]. Impairment of non-lysosomal glucosylceramidase (*Gba2*, [37]) also increased phenotypic severity in double-mutant mice. These preclinical data support the hypothesis that genetic modifiers may contribute to phenotypic heterogeneity in NPC1 disease.

Sterol O-acyltransferase 1 (SOAT1) and sterol o-acyltransferase 2 (SOAT2) catalyze the intracellular esterification of cholesterol. SOAT1 is also referred to as ACAT1; however, ACAT1 can also refer to the mitochondrial enzyme acetyl-CoA acetyltransferase. SOAT1 is ubiquitously expressed, and it is the isoform primarily expressed in the brain. SOAT2 is predominantly expressed by intestinal enterocytes and liver. Impaired function of either SOAT1 or SOAT2 appears to have a beneficial effect on the NPC1 phenotype in mouse models. Double-mutant *Soat1-/-:Npc1-/-* mice, relative to *Soat+/+:Npc1-/-* mice, have delayed development of the NPC1 disease phenotype, Purkinje neurons preservation and increased survival [38]. Consistent with its expression in liver, double-mutant *Soat2-/-:Npc1-/-* mice manifested an attenuated liver phenotype with decreased accumulation of unesterified cholesterol and decreased serum transaminase levels [39]. As noted above, although NPC1 is characterized by increased unesterified cholesterol storage, there is paradoxically a functional cellular cholesterol deficiency. Esterification of the bioavailable cholesterol by SOAT1 or SOAT2 may compound this deficiency. Thus, inhibition of intracellular cholesterol esterification may improve the cellular cholesterol deficiency and explain the decrease in phenotypic severity observed in both *Soat1-/-:Npc1-/-* and *Soat2-/-:Npc1-/-* mice in comparison to single-mutant *Npc1-/-* mice.

Several studies have evaluated associations between rs1044925, a single nucleotide polymorphism in the 3′-untranslated region of *SOAT1* (C/A), with serum lipid levels [40,41], atherosclerotic disease [42] and Alzheimer’s disease/Dementia [43,44,45]. The rs1044925 A-allele is considered protective and associated with decreased *SOAT1* mRNA expression [46]. Single-eQTL data also suggests that the rs1044925 A-allele is associated with decreased expression in multiple neuronal tissues (GTExPortal). Combining these observations with the data showing an attenuated neurological disease phenotype in double-mutant *Soat1-/-:Npc1-/-* mice, we hypothesized that the rs1044925 A-allele could be a genetic modifier of the NPC1 phenotype in individuals with NPC1.

In this paper we characterize the degree of phenotypic heterogeneity present in individuals with NPC1 with the same *NPC1* genotype (*NPC1^I1061T/I1061T^* and siblings). The existence of genetic modifiers has been proposed based on the multiple case reports describing phenotypic heterogeneity in individuals with the same *NPC1* genotype (reviewed in [12,47]; however, our data provide the first formal quantitative evidence for genetic modifiers in NPC1 disease. We also show that the *SOAT1* rs1044925 polymorphism C-allele is associated with a more severe NPC1 phenotype. Specifically, the C-allele is associated with an earlier age of neurological onset.

## 2. Results

### 2.1. Evidence for Genetic Modifiers in Individuals with Niemann-Pick Disease, Type C1

**Figure 1:**
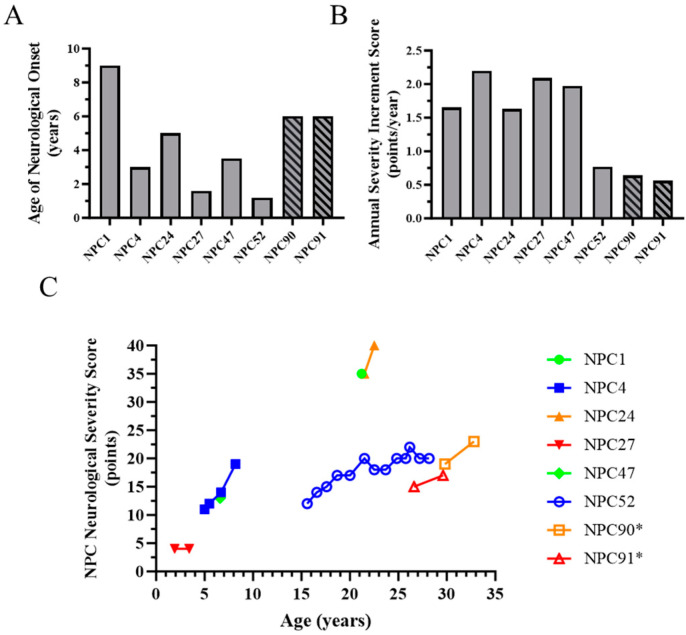
Phenotypic heterogeneity in NPC1 individuals homozygous for the p.I1061T (c.T3182C) variant. (**A**) Age of neurological onset in eight *NPC1^I1061T/I1061T^* individuals. NPC90 and NPC91 are full siblings. (**B**) Annual Severity Increment Scores in these same eight *NPC1^I1061T/I1061T^* individuals. (**C**) Longitudinal progression of the 17-domain NPC Neurological Severity Score in these eight *NPC1^I1061T/I1061T^* individuals. * siblings.

NPC1 neurological phenotypic heterogeneity can be characterized by age of neurological onset, the NPC Neurological Severity Scale (NPC NSS, [11]) and the Annualized Severity Index Score (ASIS, [48]). The NPC NSS is a Likert-like scale that assesses neurological severity in nine major and eight minor domains. Higher NPC NSS scores indicate increased disease burden. The full 17-domain NPC NSS and an abbreviated 5-domain (Ambulation, Fine Motor, Swallowing, Speech, Cognition) NPC NSS have been shown to ascertain clinically relevant aspects of the disease and measure clinically significant changes [49,50]. The NPC NSS can be used both retrospectively and prospectively to describe progression in individuals with NPC1. The ASIS age normalizes the NPC NSS. Decreased age of neurological onset and increased ASIS scores are indicative of a more severe NPC1 neurological phenotype. The most prevalent *NPC1* pathological variant in individuals of Western European heritage is p.I1061T (c.3182T>C) [51]. Although individuals who are homozygous for *NPC1^I1061T^* have the same NPC1 genotype, they manifest significant phenotypic heterogeneity. In a natural history cohort followed at the National Institutes of Health Clinical Center, we evaluated eight individuals with a homozygous *NPC1* p.I1061T genotype. Two of these individuals (NPC90 and NPC91) are siblings. Age of neurological onset varied from 1.2 to 9 years old in this cohort of individuals with *NPC1^I1061T/I1061T^* genotype (Figure 1A). Mean age of neurological onset was 4.4 ± 2.6 years. Similarly, baseline ASIS values varied from 0.56 to 2.20 points/year with a mean of 1.44 ± 0.68 points/year (Figure 1B). NPC NSS scores, a measure of increasing neurological disease burden and progression, are provided in Figure 1C. These data demonstrate the variable disease progression in individuals homozygous for the p.I1061T missense mutation.

**Figure 2:**
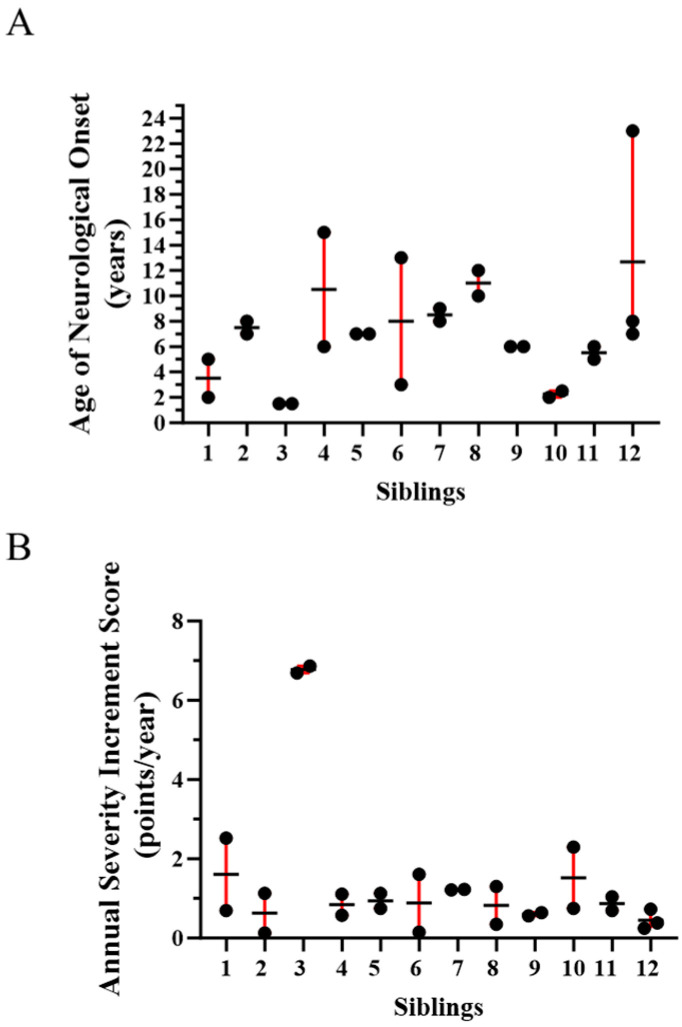
Phenotypic heterogeneity in twelve sets of siblings with NPC1. (**A**) Age of neurological onset in NPC1 siblings. Mean (horizontal bar) and range (red bar) are indicated. (**B**) Annual Severity Increment Scores in NPC1 siblings. Mean (horizontal bar) and range (red bar) are indicated.

**Figure 3:**
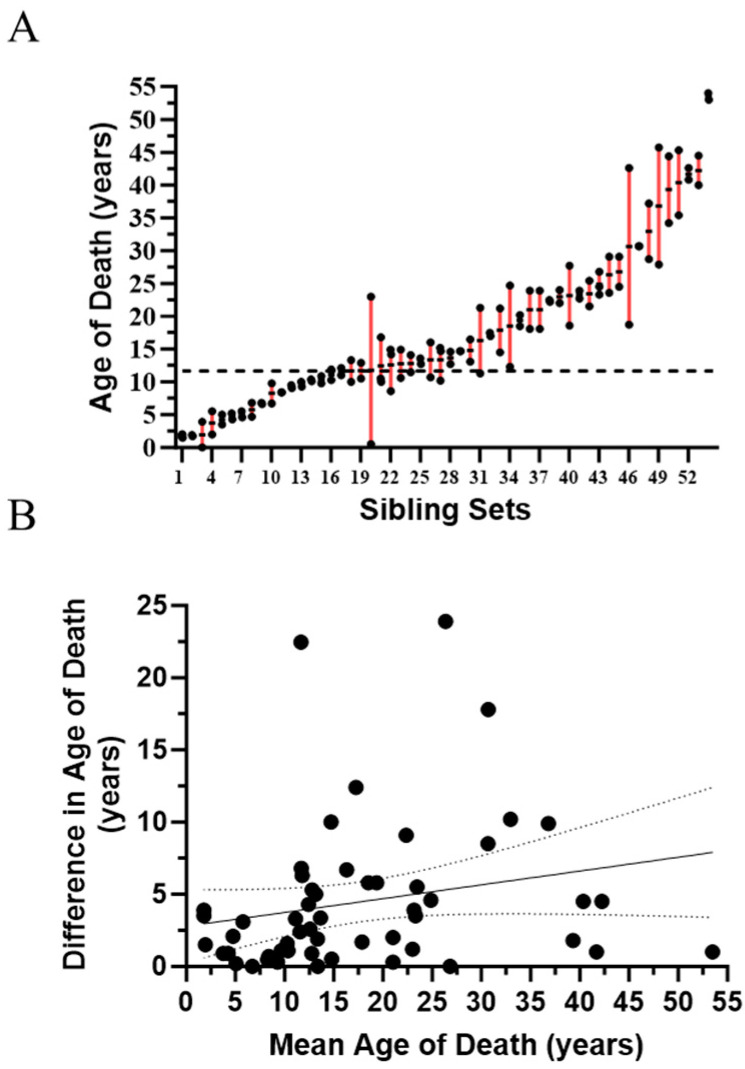
Variable age of death in 54 sets of siblings with NPC1. (**A**) Age of death is plotted for 54 sets of siblings. Range is indicated by the red bars. The horizontal dashed line is at 12 years of age. (**B**) Correlation (r^2^ = 0.05, *p* = 0.11) between mean age of death for the sibling sets and difference in the age of death. Dashed lines indicate the 95% confidence interval.

Our NPC1 natural history cohort includes 12 sibling sets manifesting NPC1 neurological symptoms. Genotypes for these sibling sets are provided in Supplementary Table S1. The “common” p.I1061T (c.3182T>C) allele accounted for approximately a third of the alleles in these siblings (8/24). A second pathological *NPC1* allele has not been identified in one of the sibling pairs; however, the NPC1 diagnosis is supported by clinical and biochemical testing. Consistent with siblings sharing half of their genome, age of neurological onset was similar (≤1.25-fold difference) in 8/12 (67%). However, the difference in the age of neurological onset ranged from 2.5- to 4.3-fold in the remaining four sibling sets (Figure 2A). We also evaluated ASIS in these sibling sets (Figure 2B). Again, ASIS was similar (≤2-fold difference) in the majority (7/12, 58%), but varied 3.1- to 10.7-fold in the remaining five sibling pairs. We were also able to obtain age of death information on 54 sets of siblings (49 pairs, four triplets, one quadruplet) from the National Niemann Pick Disease Foundation. Age of death was relatively consistent in siblings where the mean age of death was under ~12 years of age (Figure 3A). However, in the remaining 34 sibling sets (mean age of death > 12 years), the difference in the range of age of death was ≥5 years in 16 (47%) and ≥10 years in 6 (18%). The variability appeared to increase after ~12 years of age, but there was no relationship (r^2^ = 0.05, *p* = 0.11) between difference in age of death and mean age of death for the siblings (Figure 3B). Although much of these data predate the use of miglustat for treatment of NPC1, earlier diagnosis and earlier interventions could have had a positive influence on the second child and thus confound the interpretation of these data. To explore this possibility, we determined the order of death for 34 sibling pairs where the cause of death was neurological and the difference in the age of death was more than one year. The first-born sibling died at a younger age in 19/34 (56%) cases. This was not significantly different than what one would expect if order of death was random (*p* = 0.63, Chi-square). Consistent with this result, survival curves show no appreciable difference for first or second born siblings (Supplementary Figure S1A). A male/female bias was not present; 47% of the siblings were male and mean age of death for males (18.3 ± 13.1 years) and females (16.5 ± 10.8 years) was similar (Supplementary Figure S1B). These data from siblings, in combination with the data from individuals homozygous for the p.I1061T variant, quantitatively support the conclusion of significant clinical phenotypic heterogeneity in NPC1 individuals irrespective of having the same *NPC1* genotype.

### 2.2. In Vitro Cellular Phenotype Heterogeneity

**Figure 4:**
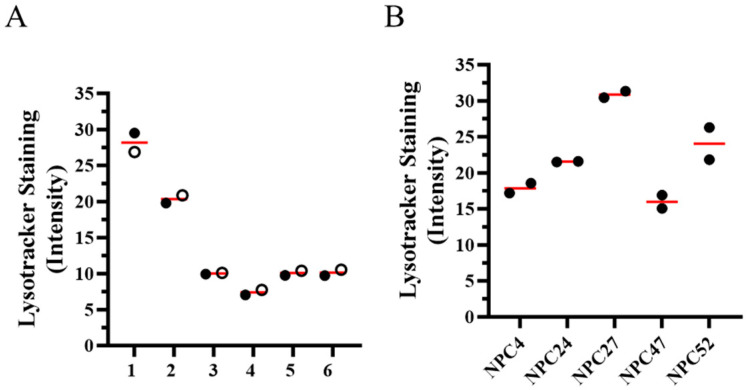
Lysotracker staining intensity in fibroblasts from sibling pairs and *NPC1^I1061T/I1061T^* individuals. Lysotracker data was previously published [53] but repurposed for this analysis. (**A**) Lysotracker staining intensity in six sibling pairs. The mean value of two measurements is indicated for each sibling as either an open or filled circle. Sibling pairs 1, 5 and 6 correspond to sibling pairs 1, 5 and 6 in Figure 2. Sibling pair 3 corresponds to sibling pair 2 in Figure 3. (**B**) Lysotracker staining intensity in five individuals homozygous for the p.I1061T variant. Two measurements from each individual, with mean value indicated by the red bar, are included.

In addition to clinical phenotypic heterogeneity, we also looked to see if the NPC1 cellular phenotype was also heterogeneous. Lysotracker staining intensity is a measure of the intracellular acidic compartment volume and is typically increased in NPC1 fibroblasts and correlated with an age adjusted severity score [52]. Lysotracker staining intensity was also positively correlated with age of neurological onset in a panel of NPC1 fibroblasts [53]. From these prior data, we were able to extract lysotracker staining intensity values for cell lines from six sibling pairs (Figure 4A) and five homozygous p.I1061T cell lines (Figure 4B). Lysotracker staining intensity was similar in the six sibling pairs; however, mean lysotracker staining fold-change (stained vs. unstained) varied 1.9-fold (16.0 ± 1.3 to 30.9 ± 0.6) in cell lines homozygous for p.I1061T.

### 2.3. SOAT1 Polymorphism (rs1044925) Genotyping in Individuals with NPC1

SNP rs1044925 genotyping and phenotypic data were available from 117 individuals (234 alleles) with NPC1. The frequencies of the reference A- and C-alleles were 0.671 and 0.329, respectively. The genotype ratio of 50 AA, 57 AC and 10 CC did not differ significantly (*p* = 0.70, Chi-square) from the expected Mendelian ratio. The allele frequency in this cohort also did not differ significantly (*p* = 0.18, Chi-square) from the expected global frequency of 0.393 and 0.606 for C- and A-alleles, respectively (https://www.ncbi.nlm.nih.gov/snp/rs1044925, release version 20230706150541, accessed on 24 January 2024).

### 2.4. The SOAT1 rs1044925 C-Allele Is Associated with a More Severe NPC1 Phenotype

**Figure 5:**
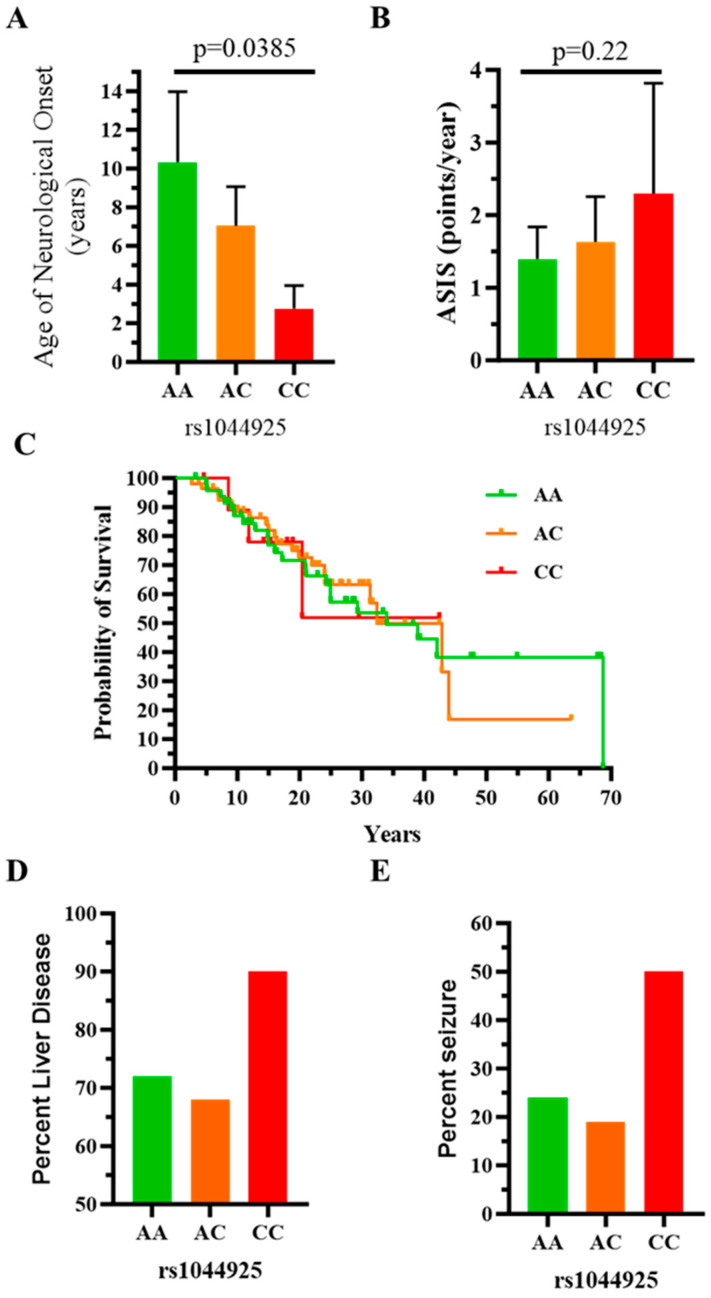
The *SOAT1* rs1044925 polymorphism C-allele is associated with a more severe NPC1 phenotype. (**A**) Age of neurological onset decreased significantly (*p* = 0.0385, Kruskal-Wallis one-way ANOVA) with the genotypic series of AA, AC and CC. (**B**) Although not significant (*p* = 0.22, Kruskal-Wallis one-way ANOVA), mean Annualized Severity Index Scores increased with the genotypic series of AA, AC and CC. (**C**) Survival curves for NPC1 individuals with the *SOAT1* rs1044925 AA (green), AC (orange) and CC (red) genotypes. Frequency of liver disease (**D**) and seizures (**E**) was higher in individuals with *SOAT1* rs1044925 CC genotype relative to individuals with either an AA or AC genotype. Mean and 95% confidence intervals are plotted in A and B.

To determine if the *SOAT1* rs1044925 polymorphism correlated with NPC1 phenotype, we stratified both age of neurological onset and baseline ASIS scores by rs1044925 genotype. The mean (95% confidence interval) age of neurological onset decreased from 10.3 (6.8, 14.0) to 7.0 (5.0, 9.1) to 2.8 (1.6, 4.0) years in individuals with AA, AC and CC genotypes, respectively (*p* = 0.0385, Kruskal-Wallis one-way ANOVA; Figure 5A). Although mean (95% confidence interval) ASIS values increased progressively from 1.4 (1.0, 1.8) to 1.6 (1.0, 2.3) to 2.3 (0.8, 3.8) points per year in individuals with AA, AC and AC genotypes, respectively, this was not significant (*p* = 0.22, Kruskal-Wallis one-way ANOVA; Figure 5B). Individual values for both age of neurological onset and ASIS are provided in Supplementary Table S2. We also explored survival in this cohort. No significant difference was observed between individuals with rs1044925 AA, AC and CC genotypes (Figure 5C). However, consistent with a more severe phenotype, the frequency of neonatal liver disease was higher in individuals with the CC genotype (90%) than in individuals with either the AA (72%) or AC (68%) genotype (Figure 5D). Similarly, the frequency of seizures was 2-fold higher in individuals with CC genotype (50%) than in individuals with either the AA (24%) or AC (19%) genotypes (Figure 5E). Genotype data for rs1044925 was available for 11 of the sibling sets. In 10/11 sibling sets, both siblings had the same rs1044925 genotype (AA n = 5 and AC n = 5). In sibling pair 6 (Figure 2), rs1044025 genotyping was AC and CC. Although one needs to be cautious about overinterpreting one example, the sibling with the CC genotype had an earlier age of neurological onset and a higher Annual Severity Increment Score. Taken in aggregate, these data are consistent with the conclusion that the *SOAT1* rs1044925 allele is associated with a more severe NPC1 phenotype.

## 3. Discussion

Phenotypic heterogeneity is a common observation in genetic disorders. Phenotypic heterogeneity can manifest both as incomplete penetrance and variable expressivity of the disease manifestations. Although environmental, microbiome and epigenetic differences clearly influence disease manifestations, variants genes, other than the disease causative gene, can play a major role. In NPC1, the *NPC1* genotype and specifically residual NPC1 function is likely the primary determinate; however, significant preclinical and clinical data supports the hypothesis that genetic modifiers significantly influence disease severity in individuals with NPC1.

In this paper, we characterized and quantified the phenotypic heterogeneity in eight individuals who are homozygous for the common p.I1061T variant and twelve sibling pairs/sets who were evaluated at the NIH. Individuals homozygous for *NPC1^I10611T/I1061T^* showed marked phenotypic heterogeneity with respect to both measures of disease severity (Age of Neurological Onset and Annual Severity Increment Score) and neurological disease burden/progression (NPC NSS). Although the longitudinal data are limited, the progression slope of the NPC NSS in the five individuals where it could be estimated ranged from 0.6 to 5.0 points/year. This supports the conclusion that the NPC1 neurological phenotype is markedly different in individuals homozygous for the common p.I1061T variant.

Similar observations were made for siblings. In general, the age of neurological onset is similar in siblings; however, marked differences were observed in a third of the sibling pairs/sets. While, in general, ASIS values are similar in sibling pair/sets, substantially different ASIS values were observed in 5/12 (~42%). We were also able to obtain survival data on 54 sets of siblings. Age of death varied widely with the difference being ≥ten years in six and ≥five years in 17 sibling sets. Variability in the age of death increased in sibling sets where the mean age of death was greater than ~12 years. Environmental factors may vary in *NPC1^I10611T/I1061T^* individuals and thus potentially contribute to phenotypic heterogeneity. However, environmental differences contributing to phenotypic heterogeneity are less likely in siblings. This variability did not appear to be related to birth order, thus decreasing the likelihood that improved care in a second child contributed substantially to the heterogeneity in survival. Although phenotypic heterogeneity has previously been noted in individuals with the same *NPC1* variants (reviewed in [12,47]), to our knowledge, the phenotypic data presented in this paper represent the largest sibling and homozygous p.I1061T cohorts quantitatively described to date, and these data clearly support the likelihood that genetic modifiers contribute to phenotypic heterogeneity in NPC1.

The effect of genetically inhibiting intracellular cholesterol esterification has been studied in both *Soat1-/-*:*Npc1-/-* [38] and *Soat2-/-:Npc1-/-* [39] mice. In both cases, it is hypothesized that inhibition of cholesterol esterification increases intracellular cholesterol bioavailability. Consistent with predominant liver expression of SOAT2, *Soat2-/-:Npc1-/-:* mice had decreased unesterified cholesterol storage in liver tissue and improved liver pathology. Similarly, consistent with expression in the central nervous system, *Soat-/-:Npc1-/-* mice, relative to *Soat+/+:Npc1-/-* mice, had delayed onset of neurological signs, improved neuropathology and significantly increased lifespans [38].

We hypothesized, based on the observation that the NPC1 phenotype is ameliorated in *Soat1-/-:Npc1-/-* mice, that functional variants in the *SOAT1* gene could contribute to genetic heterogeneity in individuals with NPC1. To test this hypothesis, we correlated the genotype of a single nucleotide polymorphism, rs1044925 (A, C), with clinical parameters of disease severity in 117 individuals with NPC1. This single nucleotide polymorphism is encoded in the 3′-UTR of *SOAT1*. The A-allele, relative to the C-allele, is experimentally associated with decreased mRNA expression [46] and single-eQTL data specifically supports decreased expression in neuronal tissues (GTExPortal). Thus, we postulated that the A-allele would be associated with a less severe NPC1 phenotype. The data in this paper show that the C-allele is significantly associated with an earlier age of neurological onset with mean age of neurological onset decreasing from 10.3 to 2.8 years of age in individuals homozygous for the A- and C-alleles, respectively. Although not statistically significant, it is notable that the mean ASIS valued increase with the genotypic series of AA, AC and CC. This is consistent with the pattern one would expect of increased NPC neurological severity being associated with the C-allele. No difference in survival was noted, but again, consistent with increased phenotypic severity being associated with the C-allele, we observed an increased percentage of individuals with liver disease and seizures in those with CC genotype. This study does have limitations that temper the conclusion. Although the cohort of individuals with NPC1 being reported in this paper is large for NPC1, numbers are still very limited when trying to establish associations between genetic variants and phenotypic findings. This limitation is inherent to the study of rare diseases and compounded in rare diseases, such as NPC1, where there is significant phenotypic heterogeneity. It is also likely that other genetic modifiers, such as ApoE isotype, contribute to this phenotypic heterogeneity. These factors impact the ability to establish statistical significance. With these caveats established, the data we present is consistent with *SOAT1* being a genetic modifier of the NPC1 phenotype, and specifically supports the idea that decreased expression of SOAT1 activity is beneficial in NPC1. This conclusion is both consistent with and substantiated by the observation of decreased phenotypic severity in *Soat1-/-*:*Npc1-/-* mice relative to *Soat1+/+*:*Npc1-/-* mice reported by Rogers et al. [38].

In conclusion, the data provided in this paper supports the hypothesis that genetic modifiers significantly contribute to phenotypic heterogeneity in NPC1 and specifically suggests that *SOAT1* is a genetic modifier of the NPC1 phenotype. By extension, our data also support the hypothesis that inhibition of SOAT1 increases intracellular cholesterol bioavailability and may have a beneficial effect in NPC1. This latter observation provides support for developing inhibitors of SOAT1 as a potential therapeutic approach for the treatment of this lethal neurodegenerative disorder.

## 4. Materials and Methods

### 4.1. Study Participants and Ethical Approval

Individuals with NPC1 were enrolled in observational studies (NCT00344331 or NCT05588167) conducted at the National Institutes of Health in Bethesda, Maryland. Ethics approval for the conduct of this clinical trial was obtained from the *Eunice Kennedy Shriver* National Institute of Child Health and Human Development Institutional Review Board or the National Institutes of Health Intramural Institutional Review Board. Written consent for participation was provided by either a guardian or the participant. When appropriate and feasible, assent was obtained.

The diagnosis of NPC1 was obtained by appropriate clinical, biochemical or molecular testing. *NPC1* genotype was obtained from clinical laboratory reports. In-depth phenotyping included medical record and history review as well as physical examination by individuals familiar with findings associated with NPC1 (NYF and FDP). Age of neurological onset was established by historical review. NPC Neurological Severity Scores were determined as previously described [11]. The full 17-domain scale was used for this study. Annual Severity Increment Scores were determined as previously described [48]. For analysis of age of neurological onset and Annual Severity Increment Scores, data from individuals who had not yet manifested NPC1 neurological symptoms was removed (n = 6). Historical data on age of death for siblings was provided by the National Niemann-Pick Disease Foundation (https://nnpdf.org/).

### 4.2. DNA Samples and Genomic Sequencing

Genomic DNA was obtained from either skin fibroblasts collected as part of a natural history study or from blood samples. Fibroblasts were cultured (37 °C, 5% CO_2_) in Dulbecco’s modified Eagle’s Medium (DMEM, ThermoFisher, Waltham, MA, USA), supplemented with 10% fetal bovine serum (FBS, Stemcell Technologies, Cambridge, MA, USA), 1X Pen/Strep and 2 mM Glutamax (ThermoFisher) in T-75 flasks. After reaching confluency, fibroblasts were isolated using 0.05% Trypsin, pelleted via low-speed centrifugation and stored at −80 °C until extracted. Genomic DNA was isolated from the fibroblast pellets using the DNeasy Blood and Tissue kit (Qiagen, Germantown, MD, USA). Whole blood samples were collected in EDTA tubes and stored at −80 °C. Genomic DNA was obtained from these samples using the QIAmp DNA Blood Midi kit (Qiagen). Quality and quantity of the DNA was determined by NanoDrop spectroscopy and the Qubit high-sensitivity dsDNA fluorescence dye binding assay kit (ThermoFisher).

Genomic sequencing was performed using the NIH Intramural Sequencing Center. Alignment was performed against human_g1k_v37 (HG19) and variants called using GAK (Broad Institute, Cambridge, MA, USA) using the “Best Practices” workflow [54], generating both single-sample VCF and GVCF files filtered as described. The presence of known SNPs was interrogated by evaluating single-sample VCF files at the given location. Average mapped coverage was ~40x and genotyping was performed for rs1044925.

### 4.3. Statistical Methods and Graphing

GraphPad Prism version 10.0.2 was used for the production of graphical images and statistical analysis.

## Data Availability

The underlying SNP genotyping, age of neurological onset and ASIS data are provided in Supplementary Table S2. Anonymized or coded clinical data is available for IRB approved research related to SLOS upon request. Genomic data has been deposited in dbGaP (phs003214.v1.p1).

## Supplementary Materials

The supporting information can be downloaded at: https://www.mdpi.com/article/10.3390/ijms25084217/s1.
