# Supplementary material for: Sterol O-Acyltransferase 1 (SOAT1): A Genetic Modifier of Niemann-Pick Disease, Type C1
Source: Int J Mol Sci. 2024 Apr 11;25(8):4217. doi: 10.3390/ijms25084217 (PMC11050712; doi:10.3390/ijms25084217)
Supplement: Supplementary file 1 [file ijms-25-04217-s001.zip › Supplemental Figure 1.pptx]

## Slide 1
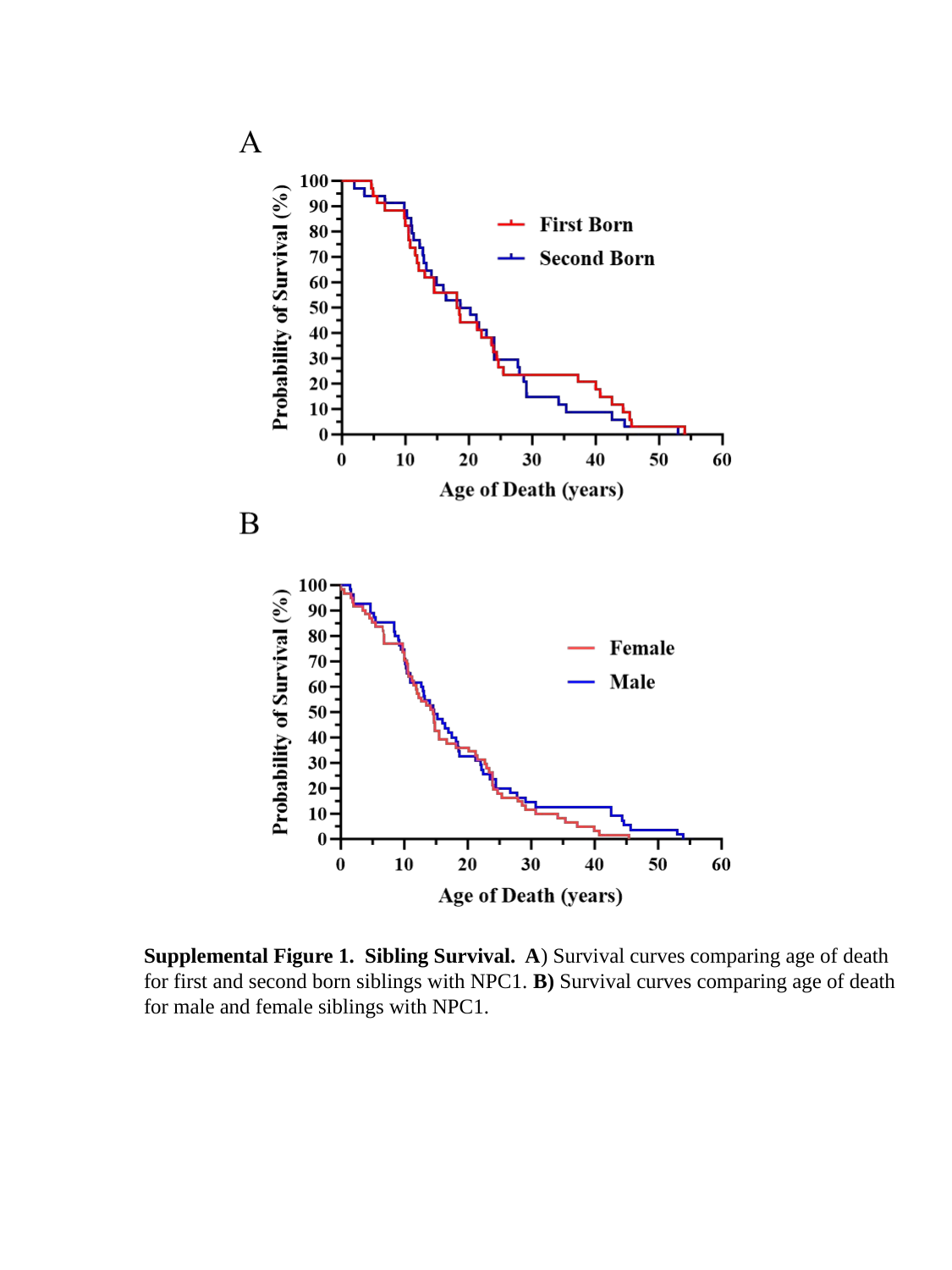

Supplemental Figure 1. Sibling Survival. A) Survival curves comparing age of death for first and second born siblings with NPC1. B) Survival curves comparing age of death for male and female siblings with NPC1.
